# Participative budgeting, job satisfaction, and presenteeism: survey evidence from middle managers

**DOI:** 10.1186/s12889-026-26572-5

**Published:** 2026-02-11

**Authors:** Berend van der Kolk, Martijn Schoute, Makoto Kuroki

**Affiliations:** 1 School of Business and Economics, Vrije Universiteit Amsterdam, De Boelelaan 1105, Amsterdam, 1081 HV The Netherlands; 2 Yokohama City University, 22-2, Seto, Kanazawa-Ku, Yokohama City, Kanagawa Prefecture 236-0027 Japan

**Keywords:** Participative budgeting, Middle managers, Wellbeing

## Abstract

**Background:**

Middle managers in large firms have demanding jobs and often experience high levels of stress. Our objective is to investigate whether and how participative budgeting can improve job-related wellbeing of middle managers. Specifically, we expect that participative budgeting has the potential to enhance perceptions of procedural justice and organizational identification, which in turn positively impact job satisfaction and diminish presenteeism, i.e., health-related productivity loss.

**Methods:**

We use survey data (*N* = 256) from middle managers of a large multinational to examine how participative budgeting (i.e., the extent to which managers are involved in deciding how and where resources are allocated) enhances procedural justice and organizational identification, and how this subsequently impacts job satisfaction. We also investigate the impact of job satisfaction on presenteeism. We use Structural Equation Modeling to analyze the relations.

**Results:**

We find that participative budgeting is positively associated with perceptions of procedural justice and organizational identification of middle managers. In turn, these perceptions are associated with higher levels of job satisfaction and lower levels of presenteeism, and thus benefit both the employee and the organization.

**Conclusion:**

We contribute to the literature by providing evidence that organizations can improve job-related wellbeing and diminish health-related productivity loss by giving middle managers a greater say in processes related to budget allocation. That is, participative budgeting enhances middle managers' perceived procedural justice and organizational identification, which subsequently positively impact job satisfaction and reduce presenteeism.

## Background

Job-related wellbeing is a matter of growing importance for both practitioners and academics [1, 2]. One group of employees who often face highly demanding workloads are middle managers, typically responsible for tasks such as implementing strategy and supervising employees in large organizations [3]. Squeezed between senior management and junior staff, middle managers "are much more likely to be overworked" than other occupational groups [4]. Recent practitioner surveys by Capterra and Microsoft indicate that 50% to 71% of middle managers sometimes or always feel stressed or burned out at work.^1^ Poor job-related wellbeing clearly impacts employees, but is also costly for organizations, negatively impacting employee retention and productivity while increasing healthcare costs [5]. Deloitte [6] estimates that in the UK alone, the costs of presenteeism—health-related productivity loss—are 24 billion GBP annually. What can organizations do to create the work conditions that improve middle managers' job-related wellbeing? Using survey data from 256 middle managers, we test whether and how participative budgeting can contribute to job-related wellbeing, and whether perceptions of procedural justice and organizational identification can help explain the underlying mechanism.

70 years ago, Argyris [7] stated that participative practices "are currently in the limelight in most management circles", and this is still true today. Involvement, increased autonomy, and influence continue to appeal to managers and organizations, and decades of research into participative work practices suggest that they come with a myriad of advantages such as enhanced performance, innovation, and creativity [8]. Such participative work practices are practices through which employees are involved in organizational decision making, and this can relate to typical tasks of middle managers such as budgeting. In this study, we focus on participative budgeting because middle managers' influence over resources is a core component of their authority. Prior research investigated the extent to which middle managers are involved in making budgeting decisions, and found this was associated with enhanced employee motivation, stronger organizational commitment, better information for decision making, and improved managerial performance [9–11]. While the (indirect) impact of participative work practices on performance has been studied extensively, research on its impact on job-related wellbeing has not always received the attention it deserves, according to critical scholars from fields such as human resources [12] and accounting [13]. Public health scholars also recently noted in a review that participative budgeting needs "a stronger evidence base" [14]. Prior research suggests that while sometimes participative work practices can increase employee wellbeing, for some employees the added challenge and work intensification may mean increased psychological strain [8, 15]. To better understand how participative budgeting impacts employees, and to extend prior research on job-related wellbeing [1, 5, 16], we study whether and how participative budgeting impacts job-related wellbeing, and how this in turn impacts health-related productivity loss.

We mobilize self-determination theory [17, 18] to formulate our expectations regarding the impact of participative budgeting on job-related wellbeing. The key tenet of self-determination theory is that employees have three needs (autonomy, competence, and relatedness), which can be addressed by organizations to different extents [19]. The better these needs are addressed, the higher employees' autonomous motivation will be, and the more employees will flourish [17, 20, 21]. Self-determination theory has been mobilized by prior research to inform expectations on participative budgeting, for instance in a survey study by Groen et al. [22] to understand the impact of participative budgeting on motivation and performance. We expect and find that participative budgeting has a positive impact on some aspects of the employee-organization relation (i.e., procedural justice and organizational identification), as they particularly address employees' needs for autonomy and competence. Furthermore, we expect and find that a positive employee-organization relation is positively associated with job-related wellbeing, as this allows employees to thrive [20].

We contribute to the literature on job-related wellbeing [1, 5, 23] by exploring how a job design choice such as participative budgeting can enhance job-related wellbeing of middle managers. Specifically, we find that participative budgeting is associated with higher job satisfaction and lower levels of presenteeism. Using self-determination theory [17], we explain the underlying mechanism by foregrounding two aspects of the employee-organization relation, i.e., procedural justice and organizational identification. We find that participative budgeting can enhance procedural justice and organizational identification, which in turn positively impact employee wellbeing. Furthermore, we add to the literature interested in the impact of participative budgeting [9, 11, 14, 22], which in the past mostly focused on performance effects [12, 13]. We show that participative budgeting can have implications for job-related wellbeing [14, 24].

The remainder of this paper is organized as follows. The next section reviews the relevant literature and develops our hypotheses on participative budgeting, the employee-organization relation, and job-related wellbeing. In section three, we explain our research method choices, and section four presents our key results and additional analyses. The last section includes a discussion situating our findings in the extant literature, our main conclusions, the limitations of our study, and suggestions for further research.

## Hypotheses development

### Participative budgeting, procedural justice, and organizational identification

This section develops hypotheses on the impact of participative budgeting on procedural justice and organizational identification.

By creating the work conditions in which employees carry out their jobs, organizations have a significant impact on job-related wellbeing [23]. Organizations increasingly adopt participatory leadership styles, which enable employees to participate in decision-making processes and allow organizations to respond quicker to environmental changes [8]. Prior research indicates that participatory leadership styles can have various positive effects for the organization, such as increased performance, creativity, and efficiency [8]. Using data from the Quality of Working Life Survey of Statistics Finland, Böckerman and colleagues [25] find that employees that are exposed to high-involvement management report higher levels of subjective wellbeing. Furthermore, participatory work practices may also result in better employee-organization relations, signalled by, for instance, higher levels of interpersonal trust and organizational citizenship behavior [26]. At the employee level, however, results are mixed. A recent literature review suggests participatory leadership styles may enhance performance and wellbeing, but may also be a source of stress for them [8]. Benolie and Somech [15] find that some participatory work practices can even be harmful to some employees. For instance, they report that for employees who score low on the personality trait conscientiousness, participatory work practices may be burdensome: "individuals who tend to be disorganized may expend more energy than necessary, and are more susceptible to psychological strain in a participative environment" [15]. In the current study, we focus on participative budgeting, as one key form of participatory work practices adopted by organizations, and study how it may contribute to job-related wellbeing.

Following self-determination theory [17, 19, 20, 27], we expect that participative budgeting can address an employee's need for autonomy and need for competence. These needs are universal necessities and form "the nutriments that are essential for optimal human development and integrity" [27]. Since these are universal needs, the extent to which an individual's needs are satisfied in the workplace can enhance intrinsic motivation, but also relates to "daily fluctuations in well-being" [27]. Applied to participative budgeting, because there is more involvement in decision making—addressing needs for autonomy and competence—employees may develop a more positive relation to their job-related activities (Ryan and Deci, 2000b), and hence, improve the employee-organization relation.

The employee-organization relation is "an overarching term to describe the relationship between the employee and the organization" [28]. While the literature has identified many dimensions of this relation [29], we will focus here on perceptions of procedural justice and organizational identification. These two aspects are not collectively exhaustive of the employee-organization relation, but comprise two important dimensions of this relation, emphasizing first the extent to which organizational procedures are perceived as fair, and second the extent to which employees identify themselves with the organization.

Procedural justice concerns the perceived fairness of the treatment of employees by the organization [30]. Although participative budgeting may also diminish procedural justice, for instance if participation is merely symbolic, without any real influence on the final budget - “pseudo-participation” [31] - or if some individuals or groups are given significantly more influence in the budgeting process than others [32], it is mostly argued to enhance procedural justice. That is, when employees are allowed to participate in the budgeting process, they perceive the process as fairer and more just. This is, among others, because a higher level of participation increases their understanding of the rationale behind budget decisions and their commitment to the budget and the organization’s goals [33, 34], which strengthens their perception that the process was fair.

Organizational identification reflects the strength of the employee's connection to the organization. Although, due to similar reasons as for procedural justice, budgetary participation may also diminish organizational identification, it is mostly argued to enhance organizational identification. That is, when employees are allowed to participate in the budgeting process, they tend to identify themselves more strongly with their organization. One of the three needs in self-determination theory is the need for relatedness, which concerns "a sense of belongingness and connectedness to the persons, group, or culture disseminating a goal" [17, 27]. This sense of belongingness can be fostered through participative budgeting, which may in turn impact organizational identification. A complementary explanatory mechanism comes from social identity theory [35], which posits that individuals derive part of their self-concept from the groups they belong to. Participation in a significant organizational process like budgeting signals to employees that they are valued members of the in-group, which reinforces their sense of belonging and strengthens their identification with the organization [36].

Following the reasoning above, we hypothesize the following related to participative budgeting, procedural justice, and organizational identification:

- H1: Participative budgeting is positively associated with procedural justice.
- H2: Participative budgeting is positively associated with organizational identification.

### The impact on job satisfaction and presenteeism

We expect that a better employee-organization relation will positively impact employee wellbeing, and the literature provides robust support for this expectation. Following self-determination theory, we expect that higher perceptions of procedural justice and organizational identification will enhance the feeling of belonging and relatedness of employees [20]. Furthermore, prior research reported that feelings of relatedness positively associate with various health and wellbeing variables [17, 20, 37].

Also outside of the self-determination literature there is ample evidence that supports the expectation that an enhanced employee-organization relation may be beneficial for employee wellbeing. For instance, McFarlin and Sweeney [38] find that procedural justice is positively associated with job satisfaction, and add that it also yields favorable organizational outcomes, such as organizational commitment and a more favorable evaluation of one's supervisor. In a longitudinal survey study, Ybema and Van den Bos [39] find that organizational justice impacts key wellbeing and health indicators; i.e., it leads to lower levels of depression and lower sickness absence of employees. Fair procedures can create a sense of trust and can lead to a more positive work experience.

Organizational identification also has the potential to impact various wellbeing indicators. For instance, using survey data from various samples including bank employees, call-centre workers and hospital employees, Van Dick et al. [40] report robust evidence of a positive association between organizational identification and job satisfaction. In addition, they also show that job satisfaction in turn strongly relates to turnover intentions of employees, rendering attention for employee job satisfaction not only a moral obligation for employers, but also a matter of concern for organizational efficiency and effectiveness.

Presenteeism concerns the "problem of workers being on the job but, because of illness or other medical conditions, not fully functioning" [59]. In other words, it is about health-related productivity loss. A substantial number of studies have tried to estimate the cost of lost productivity because of presenteeism, often estimating the cost of presenteeism for different health conditions [41, 42]. Although this is methodologically very challenging, such studies have generally shown that the costs of presenteeism are quite substantial, both in total and per health condition. Based on an extensive review of the literature, Schultz et al. [43] conclude that "health conditions are associated with on-the-job productivity losses and presenteeism is a major component of the total employer cost of those conditions, although the exact dollar amount cannot be determined at this time."We expect that higher job satisfaction reduces presenteeism, in line with prior research that reported a positive impact of wellbeing on productivity [57].

Following the reasoning and evidence presented above, we hypothesize the following:

- H3: Procedural justice is positively associated with job satisfaction.
- H4: Organizational identification is positively associated with job satisfaction.
- H5: Controlling for health status, job satisfaction is negatively associated with presenteeism.^2^

**Fig. 1:**
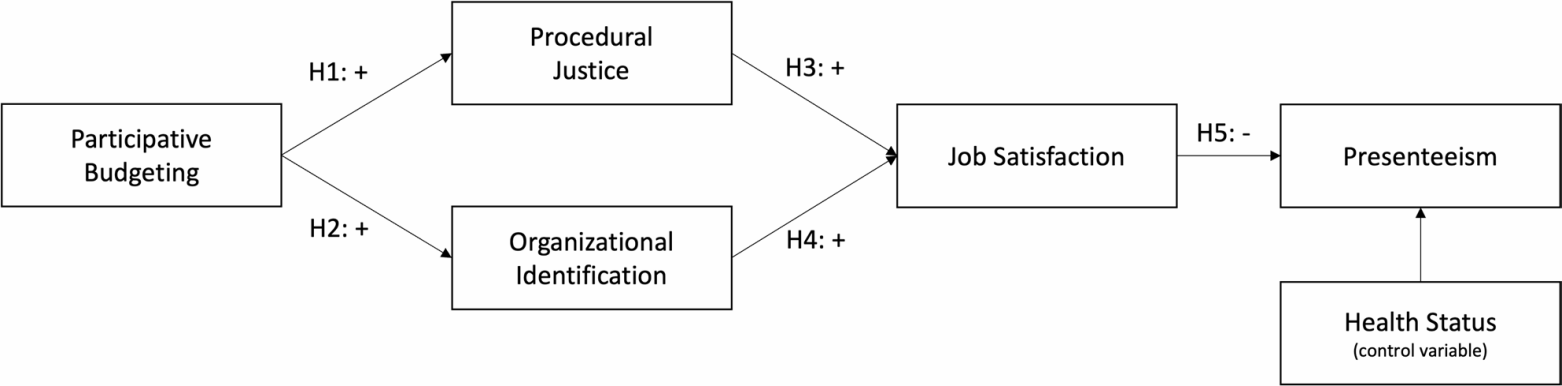
Overview of hypotheses

Our hypotheses are summarized in Fig. 1. In the following section, we discuss our research method choices, after which we will present our main results.

## Methodology

### Research site and data collection

Surveys are well suited to test hypotheses in a rich field setting, and allow the collection of responses from many participants [44]. We use survey data collected between November 18 and December 13, 2019 in a listed manufacturing firm that is headquartered in Japan: largetech (fictional name). By using responses from one organization within one country, we control for numerous confounding variables such as firm culture and national culture, which enhances the internal validity of our study. Largetech has over 30,000 employees around the world, and has been in business for over 50 years. Although largetech has subsidiaries in various countries, in this research project we only focus on responses from employees from a Japanese subsidiary to control for various internal and external factors. In this study, we focus on responses from department managers (i.e., middle managers). The research site employed a total of 506 managers in November 2019. The survey was notified to employees via email, and responses were collected through a dedicated online survey instrument, which can "increase the validity, reliability, and statistical power" and facilitates "rapid survey administration as well as data collection" [44]. A total of 256 usable responses were collected (response rate: 50.59%). Demographics of the respondents are in Table 1.

**Table 1:** Descriptive statistics sample

Panel A: Descriptive statistics for continuous variables						
**Variable**	**N**	**Mean**	**S.D**	**Median**	**Min**	**Max**
Age	237	51.7	4.4	52	41	60
Tenure	237	26.1	8.8	28	0	39
Years of education	230	14.3	1.8	14	9	18

**Panel B: Descriptive statistics for categorical variables**						
**Variable**	**N**	**Categories**			**Freq**	**Perc**
Gender	239	- Male			228	95.40%
		- Female			10	4.18%
		- Prefer not to say			1	0.42%
Education level	230	- Junior high school graduation			1	0.43%
		- High school graduation			76	33.04%
		- College graduation			5	2.17%
		- Technical college graduation			37	16.09%
		- University graduation			110	47.83%
		- Master course graduation			1	0.43%

### Measurement of the variables

To strengthen the reliability and robustness of our analyses, we used existing measures that have been shown to be unidimensional, highly reliable, and valid in earlier studies. As an ex-ante strategy to mitigate common method bias, we used a variety of scales for the different constructs.^3^ For all multi-item constructs, we specify a reflective measurement model (MM), in which observed item values are considered to be a function of the value of the latent underlying construct.

*Participative Budgeting* is measured using a validated, three-item construct developed by Kren [9], based on earlier work by Milani [10]. Respondents are asked, on a seven-point scale ranging from 1 (strongly disagree) to 7 (strongly agree), to indicate to what extent they agree with three statements that refer to the manager’s degree of participation and influence related to the budget.

*Procedural justice* is measured using a slightly adapted instrument developed by Moorman [45]. Respondents are asked, on a four-point scale ranging from 1 (strongly disagree) to 4 (strongly agree), to indicate to what extent they agree with seven statements that refer to whether they believe the procedures that are used in their organization, in particular those concerning the decision-making involving their department, are fair.

*Organizational identification* is measured using a slightly adapted instrument developed by Mael and Tetrick [46]. Respondents are asked, on a five-point scale ranging from 1 (strongly disagree) to 5 (strongly agree), to indicate to what extent they agree with five statements that refer to how strongly they identify with their organization.

*Job satisfaction* is measured using items from the Psychological Survey of Occupational Stress [47] that is developed based on Karasek et al. [48] and recommended by the Japanese Ministry of Health, Labor, and Welfare. Respondents are asked, on a four-point scale ranging from 1 (strongly disagree) to 4 (strongly agree), to indicate to what extent they agree with three statements that refer to how satisfied they are with their job.

*Presenteeism* is measured using a slightly adapted version of the validated “Stanford Presenteeism Scale” - SPS 6 [49] - consisting of six items covering performance impairments due to health problems during the past month. The SPS 6 focuses on evaluating two main dimensions: (1) Completing work, which measures the ability to finish tasks and manage workload despite health-related issues, and (2) Avoiding distraction, which evaluates the extent to which health-related issues distract from focusing on work tasks. We use the composite measure in our analysis.

*Health status* is measured using a well-established, self-reported, single-item measure from the World Health Organization (WHO), which asks the respondents to rate their current health condition on a five-point scale ranging from 1 (very poor) to 5 (very good).^4^

## Results

### Descriptive statistics and Pearson correlations

Table 2 presents the descriptive statistics and Pearson correlations based on average scores for the model variables. The correlations provide initial support for our hypotheses. As expected, participative budgeting is positively correlated with two aspects that reflect the employee-organization relation, procedural justice, and organizational identification. Both procedural justice and organizational identification are positively correlated with job satisfaction, while job satisfaction and health status are negatively correlated with presenteeism. These correlations are all consistent with our model (see Fig. 1). In addition, participative budgeting is also positively correlated with job satisfaction, which is why we will also estimate an alternative model in which we include this relation. Finally, note that the correlation between participative budgeting and presenteeism is not significant.

**Table 2:** Descriptive statistics and Pearson correlations

	Mean	S.D	1	2	3	4	5
1. Participative budgeting	4.36	1.24	-				
2. Procedural justice	2.66	0.50	0.35***	-			
3. Org. identification	3.84	0.68	0.24***	0.15**	-	-	
4. Job satisfaction	2.98	0.72	0.24***	0.31***	0.27***	-	-
5. Presenteeism	1.72	0.54	−0.00	−0.10	−0.00	0.19***	-
6. Health status	3.45	0.95	0.08	0.08	0.08	0.06	0.34***

*N =* 256, ***, **, * indicates significance at the 0.01, 0.05 and 0.10 levels (two-tailed), respectively

### Model analyses and results

We use Structural Equation Modeling (SEM) based on maximum likelihood estimation to simultaneously estimate the measurement model (relating indicators to latent constructs) and the structural model (relating latent constructs to each other). Because almost all items are measured using Likert scales, they are not normally distributed,^5^so we use the Satorra-Bentler correction procedure for the estimation of the model Chi-square and the standard errors. We use multiple fit measures to assess how well the estimated model fits the sample data and to increase the probability of rejecting a “false” model and not rejecting a “true” model [53].^6^We first discuss the measurement model estimates, and then the structural model estimates.

### Measurement model estimates

Table 3 presents descriptive statistics and measurement model estimates for all variables. We use both Cronbach’s Alpha (*α*) and McDonald’s Omega (*ω*_*h*_) to assess the reliability of the constructs. The reliability of all constructs is acceptable, as the *α*’s and *ω*_*h*_’s range between 0.79 and 0.91 [54]. To identify the scales of multi-item constructs, the loading of the indicator that was expected a priori to best represent the construct is fixed at a value of 1 [55]. To still filter out measurement error for constructs with single-item indicators (health status), we specify a subjectively determined error of 0.20 multiplied with the item’s variance [56]. The fit statistics for the measurement model show a good fit with the data, and for each construct the estimates show significant factor loadings (λ) and satisfactory standardized loadings (λs) (see Table 3).

**Table 3:** Reliability statistics and measurement model estimates

	**Panel A:** **Descriptive statistics**				**Panel B:** **Measurement model (MM)**		
	**Min**	**Max**	**Mean**	**S.D**	**λ**	***z*-Value**	**λs**
Participative budgeting (α = 0.82; ω_h_ = 0.82)							
Involved in setting all portions	1	7	4.74	1.49	0.97	12.11***	0.77
Not final until satisfied with it	1	7	3.79	1.46	0.87	12.09***	0.71
Opinion is an important factor (λ = 1)	1	7	4.56	1.38	1.00	-	0.86
Procedural justice (α = 0.86; ω_h_ = 0.86)							
Based on accurate information	1	4	2.86	0.61	0.75	10.47***	0.68
Opportunity to express opinion or raise objections	1	4	2.94	0.62	0.60	7.71***	0.53
All affected parties participate	1	4	2.49	0.72	0.95	13.11***	0.73
Consistent decision-making	1	4	2.65	0.73	1.03	13.29***	0.78
Views of all those affected are heard (λ = 1)	1	4	2.29	0.72	1.00	-	0.77
Opinions and information are collected after the fact	1	4	2.52	0.69	0.82	10.62***	0.66
Clarification or additional information can be requested	1	4	2.85	0.66	0.76	9.55***	0.64
Organizational identification (α = 0.80; ω_h_ = 0.80)							
Criticism feels like a personal insult	1	5	3.77	1.04	0.95	14.58***	0.68
Very interested in what others think	1	5	3.89	0.86	0.59	7.45***	0.51
Company's successes are my successes	1	5	3.61	0.91	0.92	15.39***	0.75
Praises feel like a personal compliment (λ = 1)	1	5	3.84	0.87	1.00	-	0.85
Criticism makes me feel embarrassed	1	5	4.11	0.87	0.61	6.85***	0.51
Job satisfaction (α = 0.91; ω_h_ = 0.91)							
Satisfied with workplace	1	4	3.00	0.78	0.93	21.81***	0.86
Satisfied with job (λ = 1)	1	4	2.97	0.78	1.00	-	0.93
Job allows to develop abilities	1	4	2.96	0.78	0.92	25.51***	0.85
Presenteeism (α = 0.79; ω_h_ = 0.80)							
No joy in work	1	4	1.68	0.76	1.05	9.67***	0.72
Difficult to deal with the stress of work	1	4	1.98	0.80	0.97	8.71***	0.64
Unable to complete the difficult tasks	1	4	1.80	0.87	1.06	8.54***	0.64
Unable to concentrate on achieving work goals (λ = 1)	1	4	1.80	0.83	1.00	-	0.63
Anxious about not being able to finish work	1	4	1.48	0.63	0.83	7.82***	0.68
Not enough energy to handle all tasks	1	4	1.56	0.69	0.63	7.11***	0.47
Health status	1	5	3.45	0.95	1.00	-	-

*N* = 256. Above are descriptive statistics and the Stata measurement model (MM) estimates for all main constructs. The MM estimates include the unstandardized factor loading (λ), significance of the loading (z-value), and standardized factor loading (λs). The MM estimates are based on ML estimation with the Satorra-Bentler estimator. Reference indicators for multi-item constructs are denoted by λ = 1. Some items (R) were reverse coded but have been reversed before the estimations. GOF statistics: *χ*^*2*^(270) = 398.14 (*p* < 0.01), RMSEA_SB = 0.04, SRMR = 0.07, CFI_SB = 0.94, TLI_SB = 0.93

### Structural model estimates

Table 4 presents the structural model estimates. The findings support our expectation that participative budgeting is positively associated with procedural justice (H1) and organizational identification (H2). The findings furthermore support the hypotheses on the positive association of procedural justice (H3) and organizational identification (H4) with job satisfaction. Together, these results indicate that as respondents experience a stronger employee-organization relation, they tend to be more satisfied with their job.

**Table 4:** Analysis of the hypothesized model

Path	Direct effect (SE)	Indirect effect (SE)	Total effect (SE)
Partic. budgeting → Procedural justice	0.19*** (0.03)	-	0.19*** (0.03)
Partic. budgeting → Org. identification	0.19*** (0.05)	-	0.19*** (0.05)
Partic. budgeting → Job satisfaction	-	0.13*** (0.03)	0.13*** (0.03)
Partic. budgeting → Presenteeism	-	−0.02** (0.01)	−0.02** (0.01)
Procedural justice → Job satisfaction	0.39*** (0.08)	-	0.39*** (0.08)
Procedural justice → Presenteeism	-	−0.05** (0.02)	−0.05** (0.02)
Org. identification → Job satisfaction	0.28*** (0.06)	-	0.28*** (0.06)
Org. identification → Presenteeism	-	−0.04** (0.02)	−0.04** (0.02)
Job satisfaction → Presenteeism	−0.14*** (0.05)	-	−0.14*** (0.05)
Health status → Presenteeism	−0.20*** (0.03)	-	−0.20*** (0.03)

*N* = 256; ***, **, * indicates significance at the 0.01, 0.05 and 0.10 levels (two-tailed), respectively. Cell statistics are the unstandardized coefficient and standard error. The estimates are based on ML estimation with the Satorra-Bentler estimator. GOF statistics: *χ*^*2*^(270) = 398.14 (*p* < 0.01), RMSEA_SB = 0.04, SRMR = 0.07, CFI_SB = 0.94, TLI_SB = 0.93

Finally, we also find support for our expectation that job satisfaction is negatively associated with presenteeism, while controlling for health status (H5). This means that as employees are more satisfied with their job, this makes them less likely to engage in presenteeism. This suggests that employees are more likely to take the necessary time off when they are ill, rather than attending work while they are less productive.

In addition to these direct effects, the indirect and total effects show that participative budgeting has a positive indirect and total effect on job satisfaction, and a negative indirect and total effect on presenteeism, within our model. This supports our idea that organizations can improve job-related wellbeing and diminish health-related productivity loss by increasing participative budgeting, and that this relation can be explained by focusing on aspects of the employee-organization relation, i.e., procedural justice and organizational identification.

### Additional analyses

We have conducted various additional analyses and robustness checks to better understand our results and the robustness of our findings.

First, given that participative budgeting is significantly correlated with job satisfaction (see Table 2), we have also estimated a model in which we add a direct effect of participative budgeting on job satisfaction. This analysis shows that this added direct effect is not significant, and that all other results are quite similar to those for our main model. This indicates that participative budgeting does not have an effect on job satisfaction, as soon as its effects via procedural justice and organizational identification are included in the model.

Second, although we do not find significant correlations between procedural justice and presenteeism, nor between organizational identification and presenteeism, we have also estimated models in which we either add direct effects of these two variables on presenteeism or replace their (indirect) effects on presenteeism via job satisfaction with direct effects on presenteeism. These analyses show that in neither of these models, procedural justice and organizational identification are significantly associated with presenteeism, and that the results for the other relations are quite similar to those in our main analysis.

Third, in addition to the main parallel mediation model, we conducted two supplemental analyses (untabulated) treating procedural justice and organizational identification as individual mediators. The results remained consistent and qualitatively similar to the main model, further validating the stability of our findings.

Fourth, to strengthen our conclusions concerning the criterion-related validity of our measure of health status, we have also estimated a model in which we add direct effects of psychological distress and the aggregate measure of chronic health issues (see end note 4) on health status (and a covariance between them) to the model. This analysis shows that both psychological distress and chronic health issues are negatively associated with health status - adding evidence of criterion-related validity for this measure - and are positively correlated with each other. Importantly, when we add these effects to our model, the results are again quite similar to our main analysis.

## Discussion

We began this paper by asking whether and how participative budgeting can positively impact job-related wellbeing, and whether perceptions of procedural justice and organizational identification can help explain the underlying mechanism. Using a large sample of middle managers from a listed multinational (*N* = 256), we find support for all of our five hypotheses. We demonstrate that participative budgeting positively impacts procedural justice (H1) and organizational identification (H2), which in turn positively associate with job satisfaction (H3, H4). Finally, we provide evidence that job satisfaction is negatively associated with presenteeism (H5). Furthermore, we show that participative budgeting also has an indirect and total effect on job satisfaction (positive) and presenteeism (negative) via two aspects of the employee-organization relation.

Our findings contribute to the employee wellbeing literature by providing evidence on the potential positive impact organizations can have on job-related wellbeing of middle managers by giving them a greater say in the budgeting processes. We extend research that seeks to understand job-related wellbeing and its antecedents [1, 5, 16, 25], by showing how participative budgeting, through enhanced procedural justice and increased organizational identification, positively impact job-related wellbeing [1, 5, 16]. Drawing on key ideas from self-determination theory [17, 20, 27], we explain how participative budgeting can enhance perceptions of procedural justice and organizational identification, which in turn impact job-related wellbeing. We help to understand how participative budgeting can positively impact job-related wellbeing by presenting evidence collected in a rich field setting, continuing the conversation on antecedents of wellbeing [8, 15]. Relatedly, our findings speak to the literature on participative budgeting. While prior research documented that participative budgeting can enhance employee motivation and improve managerial performance [9–11], its impact on job-related wellbeing received only some, but limited attention [12, 13]. By providing evidence of a relation between budgeting practices and job-related wellbeing, we extend the interdisciplinary dialogue about the psychological impact of participatory work practices. Since job-related wellbeing is associated with other outcome variables relevant to organizations, including performance and productivity [57], using participative budgeting can be beneficial for both employees and organizations.

Our findings should be considered in light of the limitations of our research method. First, while survey studies have various strengths, they also come with limitations [44]. While we have strong reasons to believe that the relations we hypothesized are indeed in the indicated directions, cross-sectional data does not allow drawing conclusions on directionality. Future research may mobilize longitudinal survey methods to provide more conclusive evidence on the direction of the described relations. Second, various biases may endanger the robustness of our findings, including common method bias and non-response bias. In our survey design and ex-post testing, we followed existing guidelines to mitigate their impact and/or indicate that we have no reason to believe they significantly impact the reliability of our findings [58]. Future research may use other methods, including longitudinal case studies, field experiments and lab experiments, which may help further unpack the relation between participative budgeting and job-related wellbeing. Third, although we followed prior research for the operationalization of our variables, we cannot rule out that the way in which we measured our main constructs comes with limitations. For instance, forms of participatory work practices other than budgeting may yield different effects, and measuring other aspects of the employee-organization relation may also result in other outcomes. Fourth, our hypotheses and models only test for linear relations, while it is not impossible that some constructs, such as organizational identification, are related to job-related wellbeing in an inverted U-shape. In other words, identifying too strongly with an organization can come at the expense of job-related wellbeing and perhaps even lead to over-commitment. We encourage future research to further examine this possibility. Lastly, we controlled for health status when we examined the relation between job satisfaction and presenteeism, but perhaps our analysis would have yielded different results if we could distinguish between different health conditions. Future research that captures more detailed information on the health status of individuals may make more precise recommendations.

## Conclusion

Using a sample of 256 middle managers from a large, listed multinational, we show that participative budgeting is positively associated with procedural justice and organizational identification, which in turn increase job-related wellbeing and diminish presenteeism. We extend the job-related wellbeing literature [1, 5, 16] by demonstrating how participation of middle managers in the budgeting process can impact job-related wellbeing. Participative budgeting and higher job satisfaction are not only beneficial for employees themselves, but also for the organizations for which they work, as prior research suggested that wellbeing and productivity are strongly related [57]. Our study helps to enhance our understanding of the intricate relation between job design choices and job-related wellbeing, while highlighting two key aspects of the employee-organization relation: procedural justice and organizational identification. We hope our findings inform interventions and policies aimed at enhancing job-related wellbeing in organizations, and inspire future research on the interplay between organizational determinants and wellbeing outcomes.

## Data Availability

The datasets generated and/or analyzed during the current study are not publicly available due to the signing of non-disclosure agreements with the participating organization. Aggregated data is available from the corresponding author on reasonable request.

## Appendix A

### Survey instrument (abbreviated)

#### Participative budgeting

Please indicate your agreement with the following statements regarding your degree of influence on your budget *(Scale: 1 = strongly disagree, 7 = strongly agree)*:

- I am involved in setting all portions of my budget.
- My budget is not final until I am satisfied with it.
- My opinion is an important factor in setting my budget.

#### Procedural justice

Please indicate your agreement with the following statements regarding the decision-making involving your department *(Scale: 1 = strongly disagree, 4 = strongly agree)*:

- Decisions are made based on accurate information.
- You are given the opportunity to express your opinion or raise objections if a decision does not go well.
- All parties affected by the decision participate in the decision.
- Decision-making is consistent (rules apply to all employees in the same way).
- The views of all those affected by the decision are heard before the decision is made.
- Opinions and information are collected after the fact about the results of decisions and implementation based on them.
- If you do not understand something about a decision, you can request clarification or additional information.

#### Organizational identification

Please indicate your agreement with the following statements regarding how strongly you identify with your organization *(Scale: 1 = strongly disagree, 5 = strongly agree)*:

- When someone criticizes our company, it feels like a personal insult.
- I am very interested in what others think about our company.
- Our company's successes are my successes.
- When someone praises our company, it feels like a personal compliment.
- If a story in the media criticized our company, I would feel embarrassed.

#### Job satisfaction

Please indicate your agreement with the following statements regarding how satisfied you are with your job*(Scale: 1 = strongly disagree, 4 = strongly agree)*:

- I am satisfied with my workplace.
- I am satisfied with my job.
- I am satisfied with my current job because it allows me to develop my abilities.

#### Presenteeism

Please indicate to what extent you have felt that your work has been affected by your mental or physical health during the past month. *(Scale: 1 = not at all, 4 = to a very great extent)*:

- The stresses of my job were hard to handle.
- I was unable to finish hard tasks in my work.
- I was distracted from taking pleasure in my work.
- I felt hopeless about finishing certain work tasks.
- At work, I was unable to focus on achieving my goals.
- I didn’t feel energetic enough to complete all my work.

#### Health status

How is your current health condition? *(Scale: 1 = very poor, 2 = poor, 3 = fair, 4 = good, 5 = very good)*

## Footnotes

1 For instance, Capterra’s middle management survey 2023 reported that 71% of middle managers sometimes or always feel stressed, overwhelmed or burned out at work. Furthermore, the Microsoft Work Trend Index 2022 among 20,000 professionals in eleven countries indicated that 53% of the managers they surveyed were burned out. Capterra: https://www.capterra.com/resources/middle-manager-burnout-strategies. Accessed on November 25, 2024. Microsoft: https://www.microsoft.com/en-us/worklab/work-trend-index/hybrid-work-is-just-work. Accessed on November 27, 2024.

2 By controlling for health status, we ensure that the impact of job satisfaction on presenteeism is robust and not confounded by the health status of individual respondents.

3 Also, a professional translation-backtranslation service (Ulatus) for English-Japanese translations was used. Survey items in Japanese were subsequently discussed and if necessary adjusted during discussions between one of the authors and LARGETECH managers.

4 This measure is also used in the National Health Interview Survey in the U.S. and the National Survey of Health and Living in Japan. Prior studies have indicated that self-reported health is related to future 'real' health status [50, 51]. We validate this single-item measure by assessing to what extent it relates to psychological distress and to five chronic health issues. We measure psychological distress using the short, well-established *Kessler Psychological Distress Scale* (K6, see [52]) and find that it is negatively associated with health status (*r* = -0.46). For the chronic health issues, we asked the respondents whether or not they have been diagnosed during a medical checkup (a) with high blood pressure, (b) with dyslipidemia (hypercholesterolemia or hypertriglyceridemia), (c) as diabetic, (d) as obese, and/or (e) had an abnormal electrocardiogram. We find that all of these chronic health issues, except (c) having been diagnosed as diabetic, are individually negatively associated with health status and that when combining them into an aggregate (count) measure (reflecting the fact that many employees had been diagnosed with multiple of these chronic health issues) the association is even stronger (*r* = -0.29). Overall, this provides strong evidence of criterion-related validity for our measure of health status.

5 Mardia’s test for multivariate skewness, Mardia’s test for multivariate kurtosis, the Henze-Zirkler test and the Doornik-Hansen test are all significant (*p* < 0.01) and thus reject the null hypothesis of multivariate normality.

6 The root mean squared error of approximation (RMSEA) and standardized root mean residual (SRMR) indicate how well the model reproduces the sample data. The comparative fit index (CFI) and Tucker-Lewis index (TLI, also called the non-normed fit index, NNFI) compare the discrepancies from a “null-model” with those from the fitted model to evaluate improvement in fit. Recommended cutoff values are 0.06 for RMSEA, 0.08 for SRMR and 0.95 for CFI and TLI, with “loosened” values for combinations of measures [53].
